# N-terminal modifications as fate switches in neurodegeneration: a mechanistic review

**DOI:** 10.3389/fnagi.2026.1809812

**Published:** 2026-06-05

**Authors:** Disha Mukherjee, Sampath Raghul Kannan, Ramasamy Tamizhselvi

**Affiliations:** School of Bio Sciences and Technology, Vellore Institute of Technology, Vellore, Tamil Nadu, India

**Keywords:** Alzheimer’s disease, Nt acetylation, Nt methylation, N-terminal modifications, post-translational modifications

## Abstract

The accumulation of aberrant proteins or their impaired clearance leads to neurodegenerative diseases (NDs). The protein amino terminus (Nt) and its modifications determine the fate of proteins and their cellular effects. Nt acetylation, Nt methylation, and Nt myristoylation are protein Nt modifications implicated in the pathogenesis of proteinopathies like Alzheimer’s, Parkinson’s, and Huntington’s diseases by regulating the protein lifespan, folding, and interaction with protein/DNA. In particular, Nt acetylation shields proteins from degradation or targets them for the same, thereby affecting their fate. Distinct enzymes catalyze Nt acetylation, Nt methylation, and Nt myristoylation, and these modifications compete for the nascent polypeptide at the ribosomal exit tunnel. Dysregulation of Nt modifications initiates the protein aggregation cascade and could potentially induce neuroinflammation and neurodegeneration. Here, we review Nt modifications and their emerging roles in the pathogenesis of NDs. Further, we highlight the crosstalk among distinct Nt modifications and explore how their convergence may shape disease vulnerability and progression.

## 1 Introduction

Neurodegenerative diseases (NDs) include a broad spectrum of diseases with multiple pathological factors impacting the nervous system by degeneration or damage of neuronal cells in the brain. Some of the major NDs are Alzheimer’s disease (AD), Parkinson’s disease (PD), Huntington’s disease (HD), Amyotrophic lateral sclerosis (ALS), and Frontotemporal Dementia (FTD), and are characterized by impaired motor and cognitive behavior that increase patient burden and the need for serious treatment strategies. Decades of research have revealed that pathogenic protein deposits, often referred to as inclusion bodies, in the neuronal tissues are a hallmark of the onset of many NDs (Hansson, 2021; Khan et al., 2023; Mathieu et al., 2020). In addition to these inclusion bodies, another common characteristic feature of most NDs is the resultant neuroinflammation. This further aggravates protein aggregation. Protein deposits are large aggregates of protein deposited in the intracellular or extracellular milieu of cells with altered structure or amount from their native form. Protein aggregates gain function through interaction with other pathological proteins, which in turn affect cellular processes and increase the severity of the disease (Hu et al., 2025). Mechanistically, protein aggregation is a multistep process initiated when proteins in non-native conformation oligomerize to form proto-fibrils (Ahanger and Dar, 2024), which leads to the formation of amyloid fibrils and longer fibrils upon self-assembly that form toxic inclusion bodies (Majid and Khan, 2023). This amyloid fibril formation is a crucial protein deposit observed in most of the NDs (Iadanza et al., 2018). Recent studies provide a deep understanding of the protein aggregation paradigm via the concept of phase transition in cells, where amyloid fibrils form via liquid–solid phase transition (Mathieu et al., 2020).

Age, genetic inheritance, mutations, environmental cues, and injury serve as key factors influencing the onset and severity of NDs. Besides familial factors influencing the protein aggregation, protein post-translational modification (PTM) also regulates the transition of native proteins into aggregation-prone toxic protein deposits highlighted in the pathology of NDs (Iadanza et al., 2018). PTMs at the amino terminal (Nt) of proteins, affecting protein stability, protein–protein interaction (PPI), protein folding, and misfolding, play a critical role in the pathogenesis of NDs (Chang, 2023; Varland et al., 2015).

Nascent proteins exiting the ribosome tunnel during translation are exposed to a variety of modifying enzymes, which potentially alter their fate. This process, known as PTMs, is an enzymatic or non-enzymatic reaction that attaches chemical moieties to a side chain of the amino acid (Liutkute et al., 2020). PTMs that occur at the N-terminus are more prevalent, and the modifications have a wide-ranging importance in the proteins’ cellular journey. Initiator methionine (iMet) excision, N-terminal acetylation (Nt acetylation), N-terminal methylation (Nt methylation), N-terminal myristoylation (Nt myristoylation), N-terminal palmitoylation (Nt palmitoylation), N-terminal propionylation (Nt propionylation), N-terminal arginylation (Nt arginylation), and N-terminal ubiquitylation (Nt ubiquitylation) are Nt modifications that proteins undergo in the cells. These Nt modifications affect all aspects of protein functions and cellular events and have implications for many diseases like cancer, neurodegenerative disease, autoimmune disease, and parasitic infections (Øye et al., 2025; Van Damme et al., 2011; Varland et al., 2015).

In this review we (i) provide a concise overview on the major Nt modifications most relevant to brain biology (Nt acetylation, Nt methylation, Nt myristoylation), (ii) map mechanistic frameworks linking Nt state to proteostasis (N-degron vs. stabilization, autophagy routing, ER/mitochondrial stress, and phase separation), (iii) synthesize disease-module evidence in Alzheimer’s, Parkinson’s, and Huntington’s diseases, and (iv) highlight crosstalk, open questions, and therapeutic outlook.

## 2 Proteins and neurodegenerative diseases: a decades-old connection

The inability of neurons to renew themselves after neuronal loss collapses the neuronal circuitry, which is reflected in impaired cognition, memory, and motor behavior that underlie ND pathogenesis. Research from the 1970s first characterized the amyloid protein deposits in the human post-mortem brain (Young, 2009). Later, the emergence of technical advances over the past five decades has brought us a long way with the discovery of many pathological hallmarks of ND from genome- to epigenome-level and molecular- to atomic-level findings (Young, 2009). Decades of investigations have constructed a framework for ND hallmarks, including protein accumulation, aberrant proteostasis, neuronal death, neuroinflammation, and defects in DNA and RNA (Wilson et al., 2023). Our focus widens from protein deposits to the phase transition paradigm in NDs. The major neurodegenerative diseases like AD, PD, HD, and dementia have characteristic protein deposits in different regions of the brain that vary from one another. An example is amyloid β (Aβ), formed from the cleavage of amyloid precursor protein (APP), leading to excessive accumulation of Aβ plaques in the brain (Toader et al., 2024; Treusch et al., 2009). Aβ accumulation is observed in the AD brain along with tau aggregates, and the latter is seen in ALS and FTD. On the other hand, α-Synuclein (α-Syn) forms aggregates in PD and dementia, while huntingtin (Htt) deposits are specific to HD (Tsoi et al., 2023). Htt mutant proteins have aggregation-prone repeats at the Nt and a protein interacting with Htt, Huntingtin-interacting protein K (HYPK) inhibits the aggregation by chaperone activity (Raychaudhuri et al., 2008). The accumulation of such toxic proteins has various aetiologies. The intermediate oligomers in the aggregation process are likely the potential neurotoxic candidates, not the mature protein deposit (Wolfe and Cyr, 2011). The sections below examine how specific Nt modifications program fate in each disease context and where those levers may be pharmacologically tractable.

## 3 N-terminal modifications

**Figure 1:**
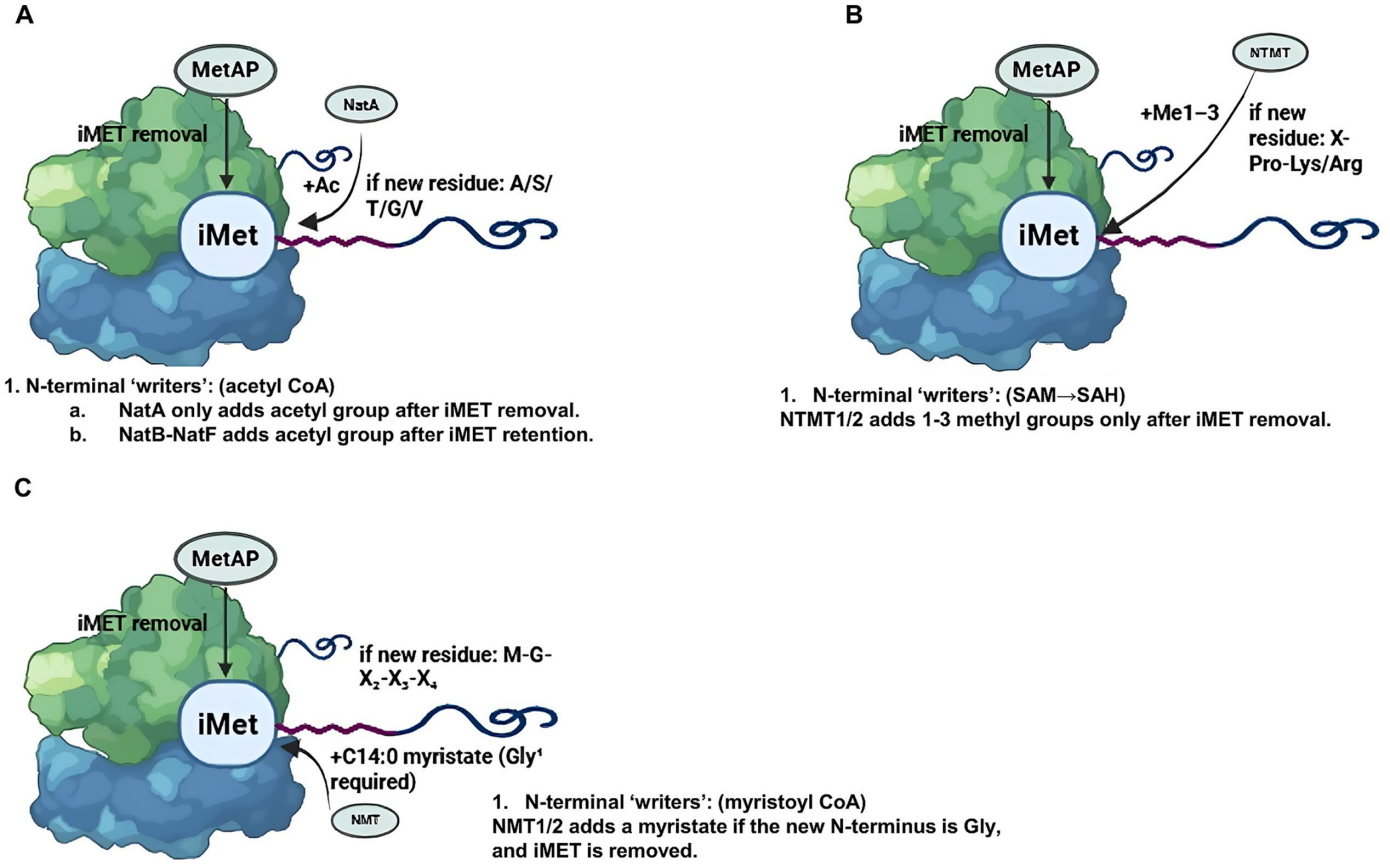
Co-translational Nt modifications. As polypeptides emerge from the ribosome, MetAP trims initiator methionine and N-terminal ‘writer’ enzymes (NATs, NTMTs, NMTs) compete for the exposed α-amine. These modifications are mostly irreversible and mutually exclusive. **(A)** Co-translational Nt acetylation. **(B)** Co-translational Nt methylation. **(C)** Co-translational Nt myristoylation.

As nascent polypeptides emerge from the ribosome, a small set of enzymes can modify the N-terminus by removing the initiator methionine and/or adding chemical groups that recode charge, shape, and binding properties at residue 1 (Figure 1). The Nt modifications include initiator methionine (iMet) excision, Nt acetylation, Nt methylation, and Nt myristoylation. These Nt modifications are mostly permanent and they play a crucial role in determining protein behavior and fate in the cell (Bailey et al., 2011). Nt modifications regulate protein folding, protein–protein interaction, protein membrane targeting, protein clearance and protein stability (Gupta et al., 2012). Ultimately, these modifications are particularly important for long-lived neurons with high proteostatic loads prone to neurodegeneration (Øye et al., 2025; Varland et al., 2015).

### 3.1 Nt methionine cleavage

N-terminal methionine excision (NME) is the earliest protein-processing event in eukaryotic cells. As the nascent polypeptide chain emerges from the ribosome, methionine aminopeptidases (MetAPs) remove the initiator methionine (iMet) co-translationally (Wiltschi et al., 2009). This reaction is strongly sequence-dependent, whereby iMet is efficiently cleaved when the second residue is small and uncharged- classically Ala, Cys, Pro, Gly, Ser, Thr, or Val, and residues further downstream can modulate efficiency (Chang, 2023; Gamerdinger and Deuerling, 2024; Giglione et al., 2004; Varland et al., 2015).

Functionally, NME sets the stage for all subsequent modifications at residue 1 (Figure 1). NME reveals a new N-terminus and allows access for other N-terminal modifying enzymes (for example, enabling NatA-mediated Nt acetylation on Ala/Ser/Gly starts, or exposing an Nt-Gly that is competent for myristoylation) (Bonissone et al., 2013). NME also determines if the N-end is read as a degradation cue or is shielded from it. In eukaryotes, N-degron pathways recognize specific N-terminal residues or chemistries and route substrates to ubiquitylation and proteasomal degradation. Conversely, capping the α-amine by acetylation, methylation, or myristoylation can mask N-end recognition. It allows for the client’s protein stabilization rather than proteasome-mediated protein degradation (Millar et al., 2019; Varshavsky, 2024). Additionally, NME subtly alters the local charge and hydrogen bonds at the extreme N-terminus. These physical changes majorly affect the downstream process, such as capping the α-amine by Nt modifications (masking N-degrons) or exposing the α-amine for N-degron machinery (Giglione et al., 2004).

Though altered NME is implicated in proteostasis and a crucial upstream regulator of several Nt modifications, direct evidence linking NME with neurodegenerative diseases is limited (Hashmi et al., 2024). For instance, Nt acetylation of α-Syn influences membrane affinity and aggregation behavior in PD models, and caspase cleavage of huntingtin can generate N-terminal fragments that become post-translationally myristoylated and rerouted for clearance (Zafar et al., 2024). Thus, even modest shifts in NME at the ribosome can affect the downstream Nt modifications that affect the stability, trafficking, and disposal of disease relevant proteins.

#### 3.1.1 Methionine aminopeptidases—the forerunner in Nt modification

Eukaryotic cytosol contains two MetAP isoforms, MetAP1 and MetAP2. Despite limited sequence homology, both share a conserved metalloprotease core flanked by regulatory regions that facilitate positioning at the ribosome exit tunnel. This allows co-translational access to the emerging N-terminus. Their substrate preferences are partially redundant: loss of either enzyme alone is often accepted, but simultaneous loss is incompatible with viability in multiple systems. This underscores the essential role of N-terminal methionine excision (NME) in proteome integrity (Chen et al., 2002; Nguyen et al., 2019). Perturbing MetAP function via genetic knockdown/knockout or even pharmacological inhibition can impact cell-cycle progression, growth, and protein stability, consistent with their placement at the very start of the protein life cycle (Chang, 2023).

From a translational perspective, MetAP2 has attracted considerable drug-discovery interest in oncology and metabolic disease, demonstrating that the active site is druggable (Grocin et al., 2021). However, given the essential nature of NME, broad inhibition is unlikely to be an immediate strategy in brain disorders. For neurodegeneration, the relevance of MetAPs lies in their gatekeeping position: by determining whether and when a nascent chain presents an acetylatable Ala/Ser/Gly start or an Nt-Gly competent for myristoylation, MetAPs indirectly condition the Nt-states of proteins such as α-synuclein, tau, APP fragments, and huntingtin. Neurons are uniquely sensitive to such early fate decisions because they host long-lived, aggregation-prone proteins and depend on tightly balanced proteostasis. Accordingly, in this review we consider MetAP1/2 as upstream regulators of the N-terminal landscape, and we discuss their downstream consequences within disease-specific modules (AD, PD, HD) and the mechanistic frameworks that couple Nt-state to proteasomal turnover, autophagy, organelle stress, and phase behavior.

### 3.2 Nt methylation

N-terminal methyltransferases (NTMTs) install one to three methyl groups on the α-amino group of a protein’s N-terminus, from S-adenosylmethionine (SAM) as the methyl donor, in a process known as Nt methylation. In metazoans, acceptor N-termini often follow a X-Pro-Lys/Arg consensus and typically arise after NME has exposed residue 2. Functionally, Nt methylation is irreversible, as no enzyme has been identified to remove α-amino methylation *in vivo* (Øye et al., 2025; Varland et al., 2015).

Methylation increases basicity and adds steric bulk at the N-terminus, strengthening electrostatic contacts with acidic partners and stabilizing certain complexes (Diaz et al., 2021; Huang, 2019). In neurons, this can modulate chromatin association of N-terminally methylated substrates like RCC1 (via NTMT1/NRMT1); and influence protein–protein and protein-DNA interactions. It can also tune N-end visibility, indirectly affecting recognition by quality-control pathways (Kats et al., 2018; Shemorry et al., 2013). Because methylation and acetylation both target the α-amino group, they can be mutually exclusive on overlapping starts; competition between Nt acetylation (shielding/degron creation) and Nt methylation (charge/affinity changes) is therefore a plausible axis controlling stability versus turnover (Section 5) and condensation versus dispersion (Section 5). Detailed, substrate-specific consequences for disease proteins are discussed in Section 5.

#### 3.2.1 N-terminal methyltransferases: three for one job

Three enzymes mediate human Nt methylation: NTMT1 (NRMT1/METTL11A), NTMT2 (NRMT2/METTL11B), and METTL13. NTMT1 is broadly expressed, allows for mono/di/tri-methylation, and recognizes the X-Pro-Lys/Arg motif with some tolerance for non-canonical flanks (Bienvenut et al., 2012; Huang, 2019). It also anchors a largely nuclear Nt-methylome that includes chromatin organizers and DNA-repair factors (Cai et al., 2014; Chen et al., 2007; Richardson et al., 2015). NTMT2 shares fold and consensus preference but is more restricted in expression and generally acts as a mono-methylase; in some contexts, it stabilizes NTMT1 engagement with non-canonical substrates. METTL13 is more distant; its best characterized activity is on eEF1A, placing it at the interface of translation and N-terminal chemistry. Regulation of NTMT abundance, localization, and cofactor supply in mammalian brain remains incompletely mapped. Where relevant, potential competition with NATs for the same starts is discussed in the disease modules.

### 3.3 Nt acetylation

Nt acetylation is the most abundant N-terminal modification in mammals. It occurs predominantly co-translationally as the nascent polypeptide chain exits the ribosome, as well as post-translationally. In metazoan proteomes, >80% of proteins acquire an acetyl group transferred from acetyl-CoA to the α-amino group by a group of enzymes called N-terminal acetyltransferases (NATs) (Ree et al., 2018). Unlike ε-lysine acetylation, Nt acetylation is generally treated as functionally irreversible *in vivo*, as no Nt deacetylase has been identified to date. The main aim is to neutralize the α-amino charge and reshape local hydrogen bonding and helix propensity, while modulating folding, protein–protein interactions, subcellular targeting, and turnover (Maze et al., 2013; Nguyen et al., 2019). Mechanistically, acetylated N-termini have two opposing roles, one by contributing to Ac/N-degron recognition and promoting protein turnover, and the other by shielding N-end degrons and stabilizing specific proteins. Again, most of these outcomes depend on amino acid sequence context, disease context, co-translational access, and partner proteins (Nguyen et al., 2018; Raghul Kannan and Tamizhselvi, 2023; Varland et al., 2023).

#### 3.3.1 N-terminal acetyltransferase: the workhorse enzyme

Eukaryotes possess at least eight N-terminal acetyltransferase complexes (NatA-NatH), where each is built around a catalytic subunit accompanied by one or more auxiliary partners responsible for assisting ribosome-proximal positioning and substrate selection (Deng and Marmorstein, 2021; Dinh et al., 2015; Starheim et al., 2012). Substrate specificity is largely dictated by the first two residues: for example, NatA acetylates N-termini beginning with Ala/Ser/Thr/Gly/Val after iMet excision, whereas other NATs recognize distinct starts or processing states (McTiernan et al., 2025). Substrate pools are overlapping but not identical; some complexes are highly specialized; for example, NatD targets histones H4/H2A, and NatH (NAA80) acetylates actin (Aksnes et al., 2019; Drazic et al., 2018). NATs are conserved across eukaryotes, and their catalytic subunits can act with or without auxiliary partners depending on the complex and context (Rathore et al., 2016). Beyond N-terminal chemistry, non-canonical roles have been reported: for example, NAA10 has been described to interact with DNMT1 and regulate DNA methylation independent of its N-terminal acetyltransferase activity (Lee et al., 2010; Ree et al., 2018). Regulation of NAT abundance, localization, and activity in mammalian brain in disease conditions remains unexplored. Genetic and cellular perturbations of NATs link NAT deletions and loss of function and mutations to diverse pathologies. Similarly, partial reduction of specific NAT activities can be beneficial in certain experimental contexts (Kabir et al., 2023; Lyon et al., 2023; Varland et al., 2023; Ward et al., 2021; Zhu et al., 2024). Thus, the cellular context is extremely important while trying to modulate NAT activity.

### 3.4 Nt myristoylation

Nt myristoylation is the covalent attachment of a C14:0 fatty acid (myristate) to the α-amino group of an N-terminal Gly (Nt-Gly). In eukaryotes, N-myristoyltransferases (NMTs) catalyze this reaction using myristoyl-CoA as the acyl donor (Wright et al., 2010). Most events are co-translational: iMet is first removed by NME, exposing Gly1, which is then acylated while the nascent chain is ribosome-proximal. A major subset is post-translational, and triggered when site-specific proteolysis (e.g., caspases, calpains) generate a neo-Nt-Gly on a pre-existing protein. Like other Nt modifications, Nt myristoylation is considered functionally irreversible *in vivo* (Martin et al., 2011).

The myristoyl group confers a weak but specific membrane affinity that is typically reinforced by adjacent basic residues (electrostatic component) and/or by secondary lipidation (e.g., palmitoylation) or partner binding. This amphitropic behavior enables conditional membrane targeting to the cytosolic leaflet of the plasma membrane, Golgi/ER, endosomes, or mitochondria and organizes signaling nanodomains, vesicle traffic, and quality-control hubs (Chang, 2023; Ree et al., 2018). Multiple ‘switch’ logics are described: (i) myristoyl-electrostatic switches, where phosphorylation or pH changes alter the charge of basic flanking residues to tune membrane residence (Ducker et al., 2005); (ii) conformational/myristoyl exposure switches (masking vs. exposure by ligand or Ca^2+^ binding) (Wang et al., 2019); and (iii) protease-gated switches, in which cleavage exposes a neo-Nt-Gly followed by myristoylation and retargeting of the fragment (Wang et al., 2021). As the myristoyl occupies the α-amino group, it is mutually exclusive with other Nt edits requiring a free α-amine (e.g., Nt acetylation, Nt methylation, Nt ubiquitylation), and often creates a competitive axis at Gly-starting N-termini.

#### 3.4.1 N-myristoyltransferases (NMTs)

Metazoans have two cytosolic NMTs, NMT1 and NMT2, with conserved active sites and partially overlapping substrate pools. Substrate engagement requires Nt-Gly and sequence features within positions 2–6, often a small/turn-permissive residue at position 3 with a basic or hydrophobic patch downstream. Co-translational access is enabled by ribosome-proximal NMTs and chaperones that present the emerging N-terminus; post-translational access depends on protease specificity and co-localization with NMTs (Wright et al., 2010). In neurons, NMT1/2 expression and compartmentalization appear differential, but brain-wide regulation and isoform-specific wiring remain incompletely mapped (Mukherjee, 2025).

## 4 Disease models

### 4.1 Parkinson’s disease (PD)

**Figure 2:**
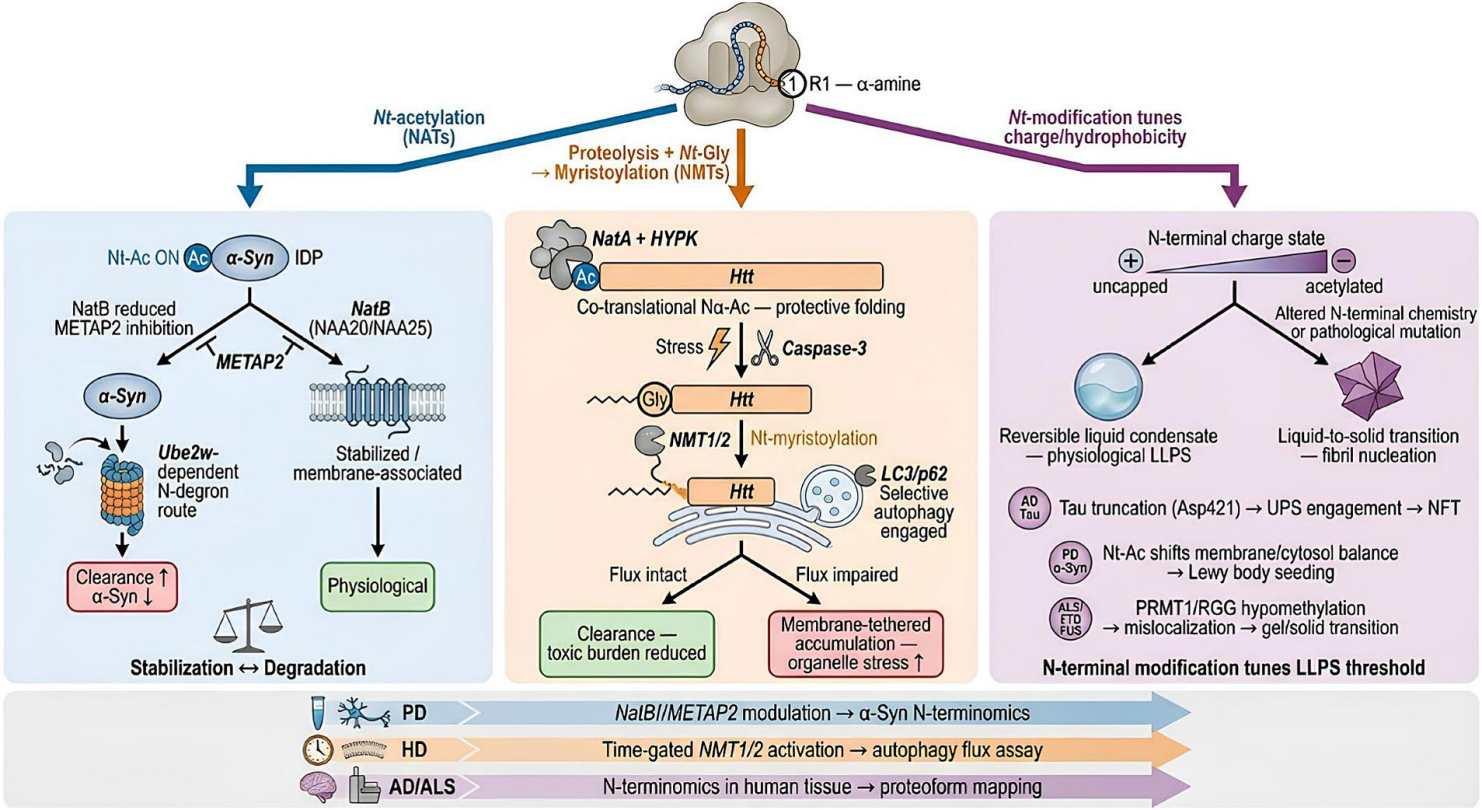
N-terminal state as a neuronal fate switch. The α-amine at residue 1 (R1) integrates enzyme access, co-translational timing, and stress-triggered proteolysis to produce three fate-determining outcomes. (Left) Degradation versus stabilization. In Parkinson’s disease, NatB-mediated Nt acetylation of α-synuclein masks its N-degron. The protein is stabilized and promotes membrane associated helical conformations. When access to NatB is reduced via METAP2 inhibition, unacetylated α-Syn is deemed as an N-degron substrate. It is cleared via a Ube2w-dependent proteasomal route which lowers baseline α-Syn levels. (Center) Stress routing and selective autophagy. In Huntington’s disease, co-translational Nα acetylation of a full-length Htt is facilitated by NatA/HYPK. Under apoptotic stress, caspase-3 cleavage exposes a neo-N-terminal glycine. NMT1/2 myristoylates the terminal glycine post-translationally. Htt fragments tether to ER-like membranes and engage LC3/p62-dependent selective autophagy. Intact autophagic flux reduces toxic burden. When impaired, membrane-tethered intermediates end up amplifying organelle stress. (Right) Condensate versus fibril. N-terminal charge and hydrophobicity tune the LLPS threshold and liquid-to-solid transition. In AD, truncation of the tau protein at Asp421 biases proteasomal participation and results in NFT formation. In PD, Nt acetylation of α-Syn modulates membrane-cytosol partitioning, paving way for Lewy body formation. Lastly, in ALS/FTD, PRMT1-dependent RGG hypomethylation of FUS engages aberrant cytoplasmic accumulation. It further allows formation of pathological gel/solid condensates. (Bottom) Experimental and therapeutic axes. Three strategies are depicted: NatB/METAP2 modulating α-Syn N-terminomic as a readout in PD; time-gated NMT1/2 activation coupled to autophagy flux assays in HD; and quantitative N-terminomic in human iPSC neurons and post-mortem brain tissue for proteoform mapping across AD and ALS/FTD.

α-Synuclein (α-Syn/SNCA; presynaptic, intrinsically disordered protein (IDP); 140 aa) is central to PD biology; LRRK2-linked signaling and proteostasis/trafficking machinery code its levels and fate (Giráldez-Pérez et al., 2014). The N-terminus (KTKEGV repeats) encodes membrane affinity and helical folding, while the nascent polypeptide-associated complex (NAC) core pushes for aggregation (Serratos et al., 2022). α-Syn begins with MDVF amino acid sequence which is a canonical NatB start. Structural work with human NatB bound to an α-Syn mimetic, along with ribosome-proximal access have shown to favor for co-translational Nt acetylation. Nt acetylation neutralizes the α-amine, increases N-terminal helicity and lipid binding, and can slow early oligomerization in simplified systems (Deng and Marmorstein, 2021; Wang et al., 2016); the same modification can also stabilize cytosolic α-Syn and change uptake, yielding context-dependent outcomes. Pooled CRISPR KO/CRISPRi screens which quantify endogenous α-Syn in human cells and induced pluripotent stem cells (iPSC)-derived neurons have scored NatB (NAA20/NAA25) among the top modifiers (Calis and Gevaert, 2025): reducing NatB activity lowers α-Syn, while non-acetylated α-Syn is cleared via a Ube2w-dependent proteasomal route to directly illustrate the N-degron vs. protection aspect (Kuo et al., 2025). Since α-Syn is mostly Nt-acetylated *in vivo*, it is mostly incompatible with α-amine-directed alternatives (Nt methylation, Nt myristoylation, Nt-ubiquitylation) at residue 1. Thus, disease-relevant shifts arise by modulating NatB access, iMet trimming, or protease-generated neo-N-termini. The clearest actionable axis so far is that partial NatB inhibition or METAP2 modulation lowers endogenous α-Syn without abolishing NatB globally (Tong et al., 2025) (Figure 2).

### 4.2 Huntington’s disease (HD)

Huntingtin (Htt) biology is unusually sensitive to its extreme N-terminus. The native protein is synthesized with the N17 amphipathic helix followed by the polyglutamine (polyQ) tract and a proline-rich region (Ghosh et al., 2025); cleavage within the N-terminal third yields fragments that dominate aggregation and toxicity. Co-translationally, the Htt nascent chain encounters the NatA-HYPK axis described previously: HYPK is a stable NatA partner at polysomes with chaperone-like properties, and multiple cell studies show that lowering HYPK or NatA increases aggregation of polyQ-containing Htt reporters (Arndt et al., 2015; Moldovean and Chiş, 2019). This places Nt acetylation, and its coupling to co-translational folding, upstream of Htt proteostasis. Mechanistically, Nt acetylation neutralizes the α-amine and can stabilize early helical structure in N17, potentially improving co-translational handling and complex assembly. At the same time, biophysical work on recombinant Htt fragments has shown that introducing the same Nt-acetyl group can accelerate oligomerization and fibrillization for specific constructs, including relatively short polyQ lengths (Adegbuyiro et al., 2017; Moldovean-Cioroianu, 2024). These observations are not contradictory when framed within the decision tree: co-translational acetylation and HYPK-assisted folding can be protective for full-length Htt and certain nascent contexts, while the similar modification on a particular proteoform (defined by fragment length, sequence around residue 1, and environment) can lower the nucleation barrier and favor assembly.

**Figure 3:**
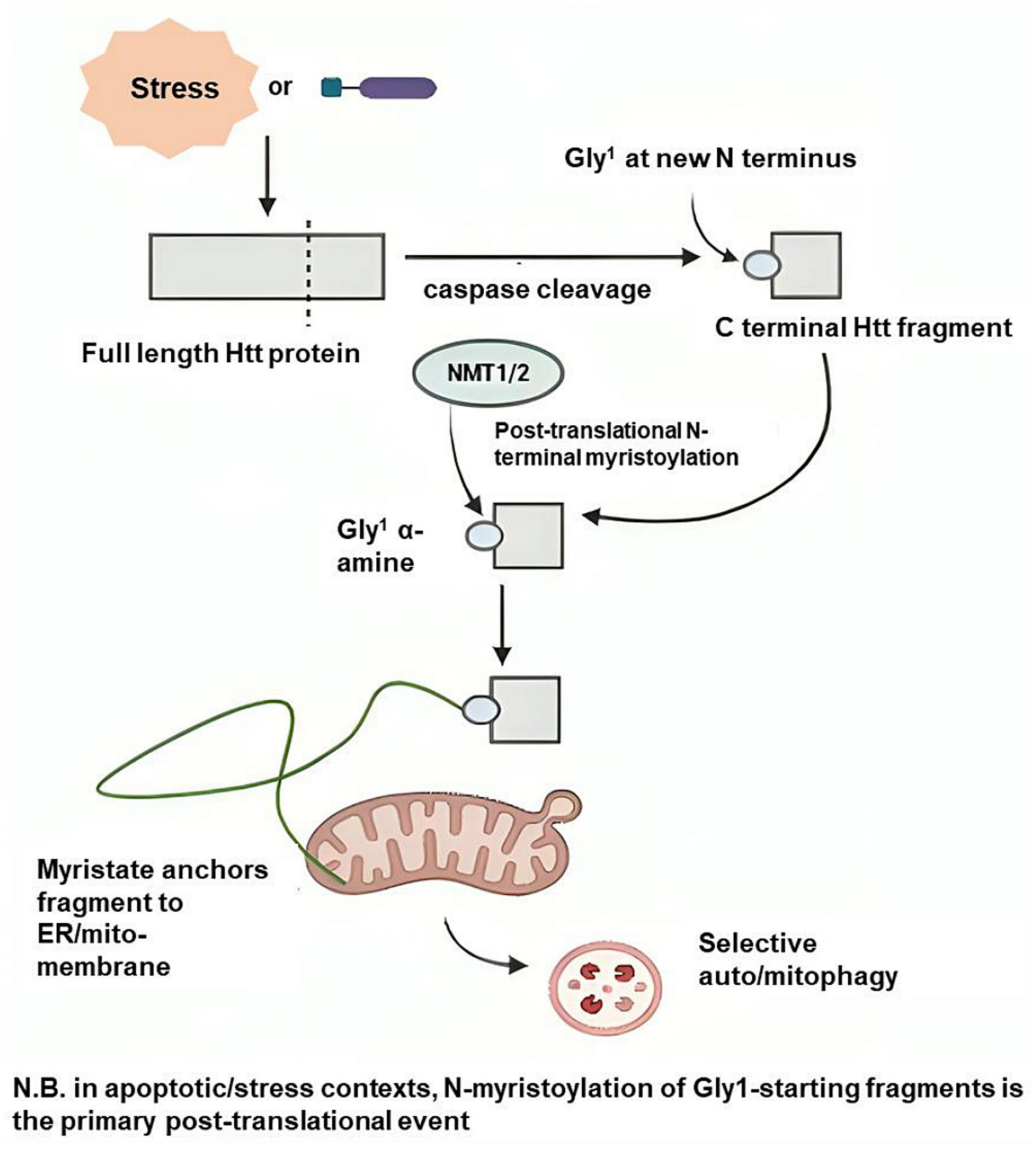
Under stress, proteases like caspases generate neo-N-termini. If the new N-terminus begins with Gly, NMT1/2 can install a myristate post-translationally. It results in retargeting the toxic fragments to membranes and allows for selective autophagy.

A second, orthogonal N-terminal lever appears during stress and apoptosis. Proteolytic cleavage is most prominently done by caspases and exposes an N-terminal Gly on specific Htt fragments (e.g., around residue 553) (Figure 3). The resulting neo-Nt-Gly licenses post-translational myristoylation by NMT1/2, which retargets these fragments to ER-like membranes and couples them to the autophagy machinery (Alshehabi and Martin, 2024). In neuronal models, myristoylated Htt (553–585) accumulates at autophagosome-forming sites and increases autophagic structures; when N-myristoylation is prevented (by mutating Gly or limiting NMT activity), this routing is lost (Mukherjee, 2025). Conceptually, the caspase–Nt-Gly–myristoyl switch provides an option for stressed neurons to redirect aggregation-prone Htt species toward membrane-organized quality-control hubs (Martin et al., 2014). However, whether this is protective depends on downstream flux: when autophagosome maturation and lysosomal fusion are efficient, retargeting reduces toxic burden. If clearance steps are impaired, membrane-tethered intermediates may instead accumulate and amplify organelle stress.

Taken together, HD illustrates the dual nature of N-terminal chemistry, which can shift in opposite directions depending on when and where it is installed, and on which proteoform it occurs. For full-length Htt at the ribosome, NatA-dependent Nt acetylation coupled to HYPK appears to support productive folding and stabilize the proteome by masking latent N-degrons (Lemarié et al., 2023). For discrete N-terminal fragments, Nt acetylation can increase assembly kinetics, shifting the balance toward oligomers and fibrils (Alshehabi and Martin, 2024). Under apoptotic or chronic stress, proteolysis followed by N-myristoylation rewires trafficking and enhances selective autophagy engagement. The clinical phenotype, particularly in striatal neurons, likely reflects the moving equilibrium among these branches of the N-terminal decision tree (mainly, co-translational stabilization versus fragment nucleation versus membrane retargeting). They are modulated by fragment spectra, chaperone capacity, lipid environment, and autophagy throughput (Figure 2).

### 4.3 Alzheimer’s disease (AD)

AD is pathologically described by extracellular Aβ plaques (derived from APP) and intracellular tau neurofibrillary tangles (NFTs) (Zhang et al., 2021). Across large autopsy cohorts without major co-pathology, higher counts of NFTs (and, secondarily, neuritic plaques) correlate strongly with worse premortem cognition, with the NFT-cognition link typically exceeding the plaque-cognition link; these associations hold even into advanced age, underscoring biological relevance rather than epiphenomenon.

APP traverses endosomes, compartments where N-end recognition and α-amine capping operate for many clients. While multiple cell-adhesion molecules modulate APP trafficking and processing, their relevance here is as indirect toggles of N-terminal exposure: by altering residence in APP-rich endosomal microdomains, they may change whether APP/β- C-terminal fragments (CTFs) N-termini are capped vs. free, potentially biasing Aβ production (Choy et al., 2012; Haass et al., 2012). Systematic N-terminomics of APP/β-CTFs in these compartments is therefore a priority test of the N-terminal framework.

*In situ* imaging and quantitative immunohistochemistry in AD brain place ubiquitin on a subset of NFTs and dystrophic neurites; mechanistically, ubiquitin colocalization is strongest with phosphorylated tau and with early C-terminal truncation at Asp421, whereas late Glu391-truncated tau shows scant ubiquitin association (García-Sierra et al., 2012). This pattern supports a timeline in which early phosphorylation following truncation at Asp421 coincides with ubiquitin targeting and proteasome engagement during early to intermediate tangle maturation (Weng and He, 2021), while later-stage Glu391 truncation is comparatively uncoupled from the ubiquitin-proteasome system (UPS) marks.

In cell systems seeded with tau fibrils, established inclusions can be reduced when soluble tau expression is suppressed, with the autophagy-lysosome pathway contributing to clearance; nonetheless, both UPS and autophagy are inefficient against large inclusions, and small residual aggregates can rapidly rekindle pathology when tau levels return (Guo et al., 2016; Kim et al., 2025). This stresses why partial or transient interventions may fail to durably reset tau burden (Drew et al., 2010). Live imaging further shows aggregates are dynamic (fusion/fission) and can propagate through cell division, a behavior consistent with the need to extinguish seed-competent species rather than only shrink bulk inclusions.

For APP/Aβ, N-terminal modifications of APP per se remain less mapped than APP-CAM/secretase control, but APP’s cell-surface context enables Nt-state to influence trafficking, partner binding, and degron exposure in other proteins. This makes Nt-rules relevant to test in APP-rich compartments (synapses, endosomes) (Zhu et al., 2024). For tau, the AD pathology timeline shows that specific proteolytic events (e.g., Asp421) emerge early and coincide with UPS labeling, while later tau forms appear less UPS-engaged (Drew and Barnham, 2011; Sun et al., 2026); this dovetails with our framework that Nt-state and early proteoform specification can bias whether a client is stabilized, routed to lysosomes, or tagged for proteasome, and can alter its propensity to enter/exit condensates. Clinically, the strong NFT/cognition correlation emphasizes that getting the tau proteoform balance right (not merely total tau levels) may be critical for therapeutic success (Figure 2).

### 4.4 ALS/FTD: TDP-43 and FUS- an N-terminal and LLPS focused view

ALS/FTD pathology revolves around the RNA-binding proteins TDP-43 and FUS, which normally shuttle between nucleus and cytoplasm, assemble into membraneless organelles through liquid–liquid phase separation (LLPS). Both the proteins are nuclear while their insoluble aggregates are cytosolic. Although both proteins carry structured N-terminal domains (NTDs) that are central to their function and disease behavior, they differ fundamentally in how N-terminal modification figures into their pathological trajectories, and this distinction deserves careful treatment (Pandey et al., 2007).

FUS (Fused in Sarcoma; 526 aa) presents a mechanistically richer landscape for post-translational modifications. Unlike TDP-43, the disease-relevant phase behavior of FUS is directly shaped by features at and near its N-terminus, making it a more tractable entry point for the Nt-modification framework (Portz et al., 2021; Qamar et al., 2018). FUS undergoes LLPS based on the Nt acetylation mediated by NatA and other post-translational modifications like lysine acetylation, methylation and phosphorylation (Dignon et al., 2018; Bock et al., 2021). FUS carries a large, intrinsically disordered N-terminal low-complexity domain (LCD; residues ~1–165) enriched in QGSY repeats, which is the primary driver of LLPS and condensate formation (Kang et al., 2019). The LCD is also the principal locus of pathological aggregation and ALS-linked mutations (e.g., R521C, R521H, R522G in the NLS; P525L) and cytoplasmic mislocalization both enhance liquid-to-solid transitions within LCD-driven condensates. Nt acetylation of FUS neutralizes the positive charge of the α-amine and alters local electrostatic properties at the N-terminus, which can modulate the charge patterning that governs FUS LCD domain in condensate formation (Khan et al., 2017; Hurtle et al., 2023). Mechanistically, Nt acetylation or other Nt modifications may reduce propensity for LLPS formation commonly observed in other proteinopathies.

TDP-43 (TARDBP; 414 aa), on the other hand, has NTD, two RNA-recognition motifs, and C-terminal domain (CTD). NTD oligomerisation mediates the LLPS while the CTD determines the nuclear-cytoplasmic shuttling and other functions (Suk and Rousseaux, 2020; Lang et al., 2024). The NTD encompasses approximately the first 80 residues, within which the extreme N-terminus (residues 1–10, including the β-strand formed by Arg6-Val7-Thr8-Glu9) contributes to dimer stability and is essential for splicing activity, including CFTR exon 9 skipping; mutations in this region abolish these functions without altering subcellular localization (Carlomagno et al., 2014; Hofweber et al., 2018; Hurtle et al., 2023; Zhu et al., 2024). However, direct evidence of N-terminal modification regulating the nuclear-cytoplasmic transport or LLPS formation is lacking.

In patient tissue and models, TDP-43 frequently shows nuclear depletion with cytoplasmic inclusions. CTFs generated by proteolysis accumulate in insoluble fractions and recapitulate cytoplasmic aggregation, hyperphosphorylation, and toxicity. Disrupting nuclear import-by NLS mutation or by impairing importin engagement- shifts TDP-43 to the cytoplasm, where it can de-mix into condensates and transition toward gels and solids under stress conditions (Mann and Donnelly, 2021; Song, 2024). Once cytoplasmic, TDP-43 and FUS enter a proteostasis fork: inclusions can be surveyed by the UPS and autophagy, but both pathways struggle with large assemblies.

Taken together, TDP-43 and FUS illustrate distinct relationships with the N-terminal modification framework. For TDP-43, NTD structural integrity and NLS-dependent import are the primary governors of localization and proteostasis, with no current evidence implicating Nt modifications; rigorous future N-terminomics will be needed to determine whether Nt-modification state influences TDP-43 proteoforms in neurons. For FUS, N-terminal charge and hydrophobicity are directly coupled to LLPS behavior, disease-relevant mislocalization and condensate material properties, and the LCD-driven condensate properties that underpin ALS/FTD pathology are responsive to the physicochemical parameters that Nt modifications tune. Thus, FUS is a priority target for future studies on α-amine-directed modification in neurodegeneration.

## 5 Crosstalk and competition at the N-terminus

All N-terminal modifications such as Nt acetylation, Nt methylation, Nt myristoylation, and Nt ubiquitylation compete for the α-amine at the first residue of a nascent polypeptide. Majority of proteins are chemically modified at the N-terminus by at least one of the Nt-modifying enzymes (Magin et al., 2017). Once the modification occurs at the N-terminus, its fate is decided, and the other modifications cannot occur on that N-terminus. The cell must select among competitive enzymes, leading to a specific modification winning under physiological conditions (Klein et al., 2026; Lentzsch et al., 2024; Varland et al., 2015). The functional consequences of tilting that equilibrium in either direction is also understudied (Aksnes et al., 2019; Klein et al., 2024). This leads to the following four mechanistically distinct questions.

First, where and when enzymes meet the nascent chain determines the fate of Nt modifications. As the nascent polypeptide emerges from the ribosomal exit tunnel, MetAPs, NATs and NMTs target the protein N-terminus. Many N-terminal modifications occur at the ribosome exit tunnel (co-translational). Not all enzymes bind to the same ribosome simultaneously due to the steric hindrance (Zhang and Zeng, 2020). It is reported that competition arises when NatE binds to the ribosomes first. It hinders the cleavage of iMet by MetAPs. Alternatively, MetAPs and NatA bind simultaneously to the ribosomes and process the Nt acetylation of NatA substrates (Van Damme et al., 2015). Second, the substrate specificity shapes the type of Nt-modification (Dong et al., 2018; Gamerdinger et al., 2025). MetAPs remove the iMet thereby exposing X_2_–X_3_… sequences, which are accessible for NatA/D/G/H (Nt acetylation), NTMT1/2 (Nt methylation), or NMT1/2 (Nt myristoylation). On the other hand, if iMet is retained, NatB/C/E/F/G or α-amine-directed ubiquitylation (Ube2w-dependent) competes. The assembly of MetAPs and the removal of iMet depend on the nature of the second amino acid (Aksnes et al., 2019). Nascent polypeptides with aliphatic or hydrophilic amino acids in the second position does not undergo NME and are targeted by NatC/E/F (Aksnes et al., 2019). Pro- and Val-starting N-termini are most accessible for Nt-ubiquitination and therefore rarely Nt-acetylated (Akimov et al., 2018).

A distinct competition has been observed in proteins starting with Gly. Global analysis found that the majority of Gly-starting protein N-termini are Nt-myristoylated and some portion of proteins coexists in Nt-myristoylated or Nt-acetylated forms (Thinon et al., 2014). This is because of the structural similarity of catalytic pockets of NAA10 and NMT enzymes. They also share partial overlap in the substrate specificity (Utsumi et al., 2001). Proteins with strong consensus sequences for NMT are myristoylated with near-complete efficiency and show little or no Nt-acetylated proteoform under normal conditions (Giglione and Meinnel, 2022; Meinnel et al., 2020). Those with weaker NMT consensus sequences exhibit mixed populations in which the Nt-acetylated form can be detected alongside the myristoylated form, particularly when myristoyl-CoA is limiting or NMT activity is reduced (Castrec et al., 2018; Su et al., 2021). The coexistence of both Nt myristoylation and Nt acetylation is not merely a biochemical curiosity, rather it generates functionally distinct proteoforms from a single gene (Gamerdinger et al., 2025; Ree et al., 2018). The Nt-myristoylated form carries membrane affinity and can engage myristoyl-electrostatic switches, myristoyl-exposure switches, or protease-gated retargeting mechanisms. On the other hand, Nt-acetylated form, is cytosolic, may be subjected to Ac/N-degron recognition by E3 ligases, and follows a stability and turnover trajectory entirely distinct from its myristoylated counterpart (Etherington et al., 2023; Omoniyi et al., 2025).

The priority of Nt myristoylation or Nt acetylation may depend on the limiting availability of myristoyl-CoA or acetyl-CoA, ribosome-proximal sequestration of the emerging chain by chaperones, or competing co-translational folding events that sterically occlude the Gly α-amine (Mueller et al., 2021; Van Damme, 2021). The outcome is therefore governed not only by intrinsic enzyme affinities or substrates, but also by the kinetic mechanism at the ribosome and co-factors availability (Deng and Marmorstein, 2021; Matsumoto et al., 2023). The functional consequences of this competition are especially acute in neurons, where metabolic state, lipid availability, and stress-induced proteolysis all shift the balance (Li et al., 2025). Declining myristoyl-CoA under energetic stress or fatty acid restriction reduces NMT flux, potentially increasing the fraction of Nt-acetylated Gly-starting proteoforms and altering their membrane targeting, signaling scaffold assembly, and susceptibility to proteasomal clearance (Ree et al., 2018; Wen et al., 2019). In the context of neurodegeneration, several disease-relevant proteins contain caspase cleavage sites that generate neo-Nt-Gly residues post-translationally most prominently huntingtin fragments as described in Section 4.2- and the efficiency of post-translational myristoylation on these neo-termini is itself subject to competition with Nt acetylation if NAT complexes access the free α-amine before NMT1/2.

Third, partner availability or assembly also can tune the outcome. Among the Nt-modifying enzymes, each NATs have subunits which could alter its function. It was recently shown that NAA10 without in complex with HYPK, binds tightly to ribosomes, limiting the enzymatic turnover (Ehrnhoefer et al., 2018). When HYPK is bound to NAA10, it acts as ribosomal exchange factor by modifying the ribosomal interaction kinetics and enables the dissociation of NAA10-HYPK complex from ribosome for multiple turnovers (Lentzsch et al., 2025). Besides, depleting NatA subunits suppress HYPK levels, and depleting either HYPK or NAA10 increases aggregation of sensitive substrates, showing that N-capping and protein function are intertwined (Arnesen et al., 2010). Thus, fluctuations in NAT complexes, NTMTs, NMTs, NAC/importins, or their positioning at polysomes can tilt the same sequence toward capping (stabilization/masking) or exposure (degron visibility).

Fourth, stress rewires the queue. Under proteotoxic or apoptotic stress, caspases cleave proteins to create neo-N-termini, generating an Nt-Gly post-translational myristoyl switch. This switch retargets the fragments to membranes where autophagy/mitophagy is organized (Hanna et al., 2023). Caspases also process components of the autophagy machinery, shifting the autophagy—apoptosis balance (Wu et al., 2014). This means the same protein can move from an acetylated, stable proteoform in homeostasis to a cleaved, myristoylated, membrane-tethered proteoform under stress, symbolizing a routing flip driven entirely by N-terminal state (Alshehabi and Martin, 2024).

Therefore, the consensus sequence of amino acid at the N-terminal determines which enzyme and modification should occur at the N-terminus (Hanna et al., 2023; Pokrzywa et al., 2017). However, the final outcome is not set in stone. The N-terminus can be thought of as a molecular logic gate at residue 1. Similar to tandem riboswitches that compute Boolean outcomes from ordered sensors, the N-terminus integrates input layers, from sequence motif (X_2_–X_3_), co-translational proximity, cofactor/enzyme availability, to stress-triggered proteolysis, producing a digital decision: cap (Ac/Me/Myr) vs. expose (degron/Ub), membrane vs. cytosol, condensate vs. diffuse, clearance vs. persistence (Romero et al., 2025; Sherlock et al., 2018, 2022; Stephen et al., 2025). Thinking of the N-end as a serial, AND/OR-like gate is useful for predicting which proteoform will dominate in neurons where local concentrations and timing are extreme. This idea may be especially relevant in contexts where local translation or stress-induced proteolysis can shift the balance between competing N-terminal states. Resolving this competition experimentally by using quantitative N-terminomics capable of distinguishing Nt-acetyl from Nt-myristoyl marks on endogenous proteins in brain tissue is therefore, an important methodological priority for the field.

## 6 Therapeutic outlook and future perspectives

Taken together, the previous sections argue for a cautious but testable principle: in general, nudging the N-terminus toward early disposal or correct routing is likely to help. For Parkinson’s disease, evidences so far indicate that α-Syn is Nt-acetylated under physiological conditions and is resistant to aggregation compared to its non-acetylated counterpart (Bartels et al., 2011; Iyer et al., 2022). Modulation of NatB activity or blocking Nt acetylation by mutating Nt sequence of α-Syn reduces α-Syn levels and neurotoxicity by targeting them for proteasomal degradation (Vinueza-Gavilanes et al., 2020). Thus, we propose that partial modulation of NatB/MetAP2-axis would reduce the pathogenic aggregation and clearance of α-Syn, without affecting the global NatB-mediated Nt acetylation.

For Huntington’s disease, during stress, caspase cleavage can expose an Nt-Gly on specific huntingtin fragments. It paves the way for post-translational Nt myristoylation. Nt-myristoylated Htt fragments get redirected to endomembranes and couples to the autophagy machinery for clearance (Alshehabi and Martin, 2024). In cell models, a myristoylated HTT553–586 fragment localizes to ER-like membranes, induces autophagosome formation, and accumulates in autophagolysosomes, linking apoptotic processing to membrane organized quality control. This suggests that NMT1/2 modulation could be explored as a way to bias clearance again, a hypothesis that needs neuronal systems and *in vivo* validation.

More broadly, NATs, NTMT1/2, and NMT1/2 are the primary Nt-modifying enzymes considered here. The Nt modification landscape is shaped by a delicate balance among MetAPs, NATs, NMTs and NTMTs. MetAPs and NATs work in combination to decide which NAT should acetylate, while NMTs come into play after caspase cleavage generating neo-N-terminus. Further, several Gly-starting N-termini are prone for Nt myristoylation as well as Nt acetylation. Beside the Nt-modifying enzymes discussed here, there are several enzymes which engage in Nt-modification. Furthermore, some PTM enzymes work in tandem to decide the functionality or property of proteins. The current cutting-edge technologies like cryo-EM and advanced proteomic studies should explore the translating ribosome and protein Nt to uncover how different Nt modifying enzymes assembly at the protein Nt and perform their functions. Such studies would contribute to our understanding of competition at protein Nt and also would further contribute for discovery of new Nt modifying enzymes. Blocking the action of one enzyme would affect the Nt-modification of proteins by altering the function of other Nt-modifying enzymes. For instance, systemic blockade or inhibition of MetAPs would hinder the NatA/D/G/H-mediated Nt acetylation and NTMTs-mediated Nt methylation. Currently, NMT inhibitors are under clinical trials for cancer treatment and NatA/D inhibitors have been proposed for cancer treatment. However, the efficiency of the inhibitors and how far it could be useful in the disease treatment without affecting the physiological processes is yet to be investigated. Moreover, the enzymes responsible for reversing Nt modifications remain unidentified, raising fundamental questions about the regulatory mechanisms that govern these modifications.

Building on this interconnected enzymatic framework, the question of selective Nt-modification and neuronal susceptibility deserves particular attention. Different neuronal populations show strikingly non-uniform vulnerability in each neurodegenerative disease: dopaminergic neurons of the substantia nigra pars compacta in PD, striatal medium spiny neurons in HD, hippocampal and entorhinal cortical neurons in AD, and upper and lower motor neurons in ALS. The N-terminal modification framework offers a mechanistic hypothesis for why this selectivity might arise. Neurons differ substantially in their protein expression, metabolic profiles, lipid compositions in different brain regions. Additionally, local concentrations of acetyl-CoA and myristoyl-CoA, expression levels of specific NAT and NMT isoforms, and the abundance of chaperone partners such as HYPK that tune co-translational N-capping efficiency also varies in neurons. These cell-type-specific differences could bias the N-terminal modification landscape of shared substrates (e.g., α-synuclein, tau, or TDP-43) in ways that makes certain neuronal populations particularly vulnerable to proteoform misregulation.

Mapping Nt-modification states across neuronal subtypes by using region-specific N-terminomics in human post-mortem tissue and in cell-type-resolved iPSC-derived neuronal populations, is therefore a high-priority avenue for understanding why certain neurons die first and others are spared. Therapeutically, the implication is not to chase one universal modifier but to nudge the N-terminal decision tree toward early disposal or correct routing without collapsing global proteostasis. From a purely methodological perspective, this field has the potential to employ enrichment mass spectrometry for native N-termini, live-cell reporters of UPS/autophagy-lysosome pathway (ALP) flux, LLPS material-state assays, as well as human iPSC-derived neurons/organoids, to map these switches accurately across species, brain regions, and disease stages (Monzio Compagnoni et al., 2018).

**Figure 4:**
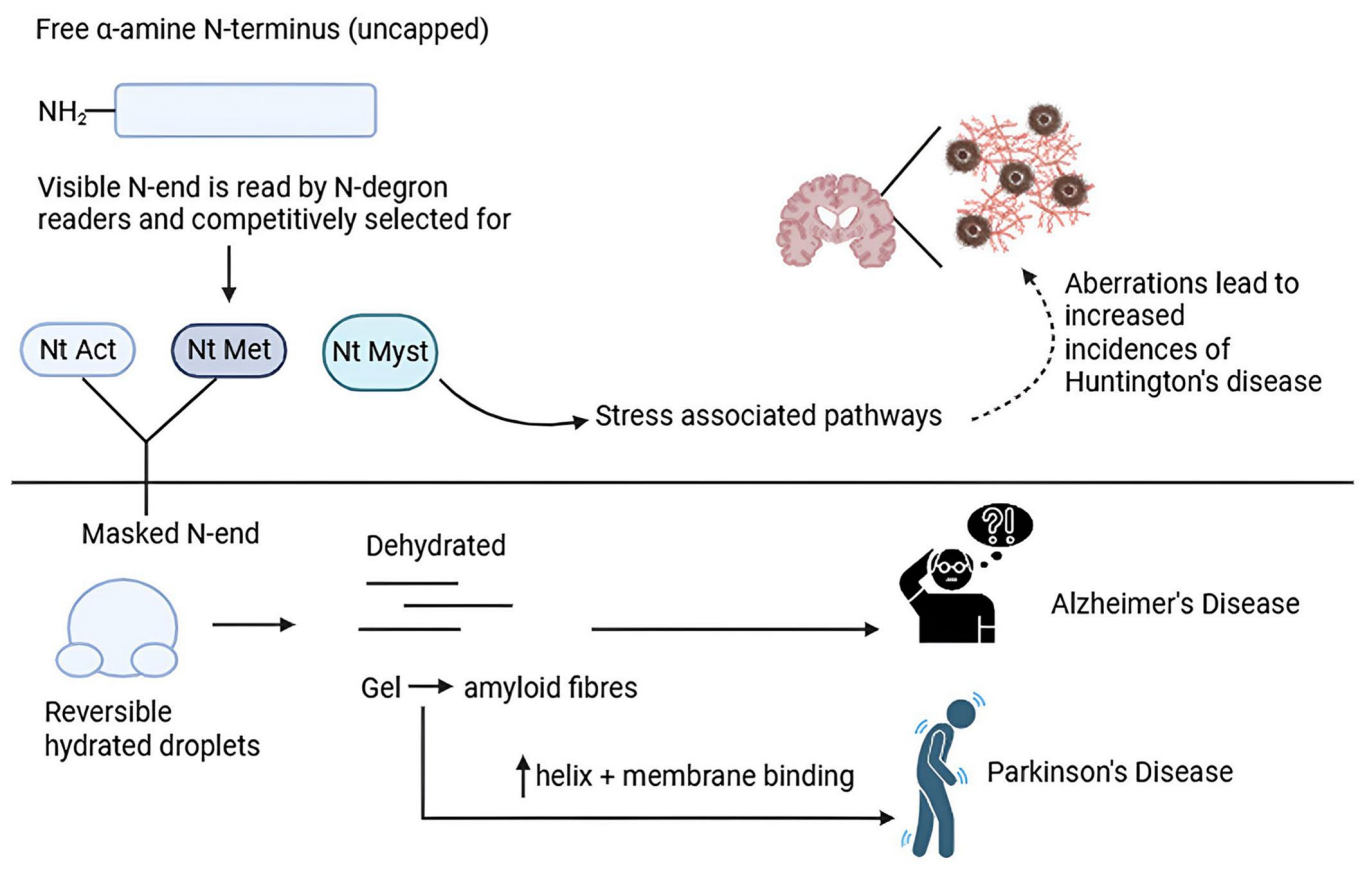
N-degron pathways reads the free α-amine (uncapped N-end) and promotes ubiquitylation and proteasomal degradation. Capping the same α-amine by Nt acetylation (NATs), Nt methylation (NTMTs), or Nt myristoylation (NMTs) is mutually exclusive. Degron recognition is halted and redirects the associated molecules to membranes. The end result is a disruption in the steady state, with a bias toward autophagy, truncation, or even pathogenic LLPS formation.

To conclude, the therapeutic premise in Figure 4, is mechanistically grounded but still unexplored. The NatB/MetAP2 axis for α-syn, stress-window N-myristoylation for Htt fragments, and exploratory NTMT1/2 probes for neuronal stress handling are reasonable paths to test, provided they are prosecuted with CNS-savvy chemistry and N-terminomics as a hard pharmacodynamic readout (Berillo et al., 2021; Chung et al., 2020). What will ultimately make these approaches credible is not a single assay but convergence: measurable shifts in N-terminal state on endogenous clients in brain models, coherent improvements in clearance and seed biology, and absence of global proteostasis penalties.

## 7 Concluding remarks

Proteins undergo N-terminal modifications, which determine their fate in the cells. Nt modifications shield the proteins from degradation, target them for protein degradation, localize proteins to intracellular organelles, cause protein aggregation, or allow protein–protein interaction. Notably, Nt modifications affect the protein folding/misfolding, protein aggregation, and clearance in neurodegenerative diseases. The type of Nt-modification and its consequences depend on the substrate specificity and disease context. Mechanistically in Parkinson’s disease, Nt acetylation provides protective mechanism by reducing aggregation of α-Syn (Nt-acetylated) and clearing α-Syn (non-acetylated) while Nt myristoylation of HTT direct them for autophagosomal degradation, indicating that modulating NatB and NMT activity could be beneficial.

All the above Nt modifications are stable and irreversible. Yet, how different Nt modifications converge or compete on shared substrates remains an unresolved mechanistic gap, and this interplay may critically shape selective neuronal susceptibility and disease progression. Interestingly, there is an interplay among the Nt-modifying enzymes, which compete for the N-terminus. The priority or selectivity of specific Nt modification greatly depends on the amino acid sequence at the Nt, enzyme kinetics, binding partner, and disease context. These parameters maybe the reason why certain proteins undergo Nt modifications while others remain unmodified. This selection ultimately shapes protein stability, localization, and aggregation propensity, thereby influencing protein homeostasis in cells and disease outcomes.

Thus, viewing N-terminal modifications as fate switches advances ND biology in two ways, firstly, it integrates proteostasis, organelle stress, and condensate physics into a single, and testable framework. Secondly, it operationalizes therapy, defining measurable, mechanistically grounded endpoints that can be met in neurons and *in vivo*. Future studies need to explore N-terminal states of proteins in human tissue especially in brain, across aging and disease. This will provide comprehensive understanding of how Nt modifications of proteins are linked to neurodegenerative diseases. The hope is that the N-terminus will shift from an overlooked peptide edge to a core control knob for neurodegeneration.
